# Quality Control of Quantitative High Throughput Screening Data

**DOI:** 10.3389/fgene.2019.00387

**Published:** 2019-05-09

**Authors:** Keith R. Shockley, Shuva Gupta, Shawn F. Harris, Soumendra N. Lahiri, Shyamal D. Peddada

**Affiliations:** ^1^Biostatistics and Computational Biology Branch, National Institute of Environmental Health Sciences, National Institutes of Health, Durham, NC, United States; ^2^Statistics Department, University of Pennsylvania, Philadelphia, PA, United States; ^3^Social and Scientific Systems, Durham, NC, United States; ^4^Department of Statistics, North Carolina State University, Raleigh, NC, United States; ^5^Department of Biostatistics, Graduate School of Public Health, University of Pittsburgh, Pittsburgh, PA, United States

**Keywords:** ANOVA, clustering, concentration-response, potency, quantitative high throughput screening, toxicological response

## Abstract

Quantitative high throughput screening (qHTS) experiments can generate 1000s of concentration-response profiles to screen compounds for potentially adverse effects. However, potency estimates for a single compound can vary considerably in study designs incorporating multiple concentration-response profiles for each compound. We introduce an automated quality control procedure based on analysis of variance (ANOVA) to identify and filter out compounds with multiple cluster response patterns and improve potency estimation in qHTS assays. Our approach, called **C**luster **A**nalysis by **S**ubgroups using **ANOVA** (*CASANOVA*), clusters compound-specific response patterns into statistically supported subgroups. Applying *CASANOVA* to 43 publicly available qHTS data sets, we found that only about 20% of compounds with response values outside of the noise band have single cluster responses. The error rates for incorrectly separating true clusters and incorrectly clumping disparate clusters were both less than 5% in extensive simulation studies. Simulation studies also showed that the bias and variance of concentration at half-maximal response (*AC_50_*) estimates were usually within 10-fold when using a weighted average approach for potency estimation. In short, *CASANOVA* effectively sorts out compounds with “inconsistent” response patterns and produces trustworthy *AC_50_* values.

## Introduction

In 1978 the National Toxicology Program (NTP) was established to evaluate the toxicity and carcinogenicity of environmental chemicals. As part of these efforts, the NTP developed a 2-year rodent cancer bioassay to identify potential human carcinogens. After about 40 years conducting such studies, the NTP has conducted evaluations for about 600 chemicals. However, over 80,000 compounds are registered for use in the United States, and that number is increasing by an estimated 2,000 new chemicals each year (U.S. National Toxicology Program [U.S. NTP], 2017). A large number of these chemicals have unknown effects on human health. Therefore, during the previous decade the NTP and other agencies, including the U.S. Environmental Protection Agency (EPA), the National Center for Advancing Translational Sciences (NCATS), and the U.S. Food and Drug Administration (FDA), established quantitative high throughput screening (qHTS) assays simultaneously screen 1000s of compounds and prioritize chemicals for further testing (Tice et al., 2013). The goal of these qHTS assays was not only to achieve the speed of evaluating 1000s of chemicals in a single experiment, but also to substantially reduce the costs of toxicity testing and, eventually, to transform toxicology into a more predictive science (Collins et al., 2008).

Quantitative high throughput screening of 1000s of different compounds at multiple concentrations represents a marked technological advancement that minimizes the frequency of false negative calls compared to single concentration HTS (Inglese et al., 2006). Data generated from qHTS have a prominent role in toxicological assessment and drug discovery (Collins et al., 2008; Roy et al., 2010; Attene-Ramos et al., 2013; Dahlin et al., 2015). For instance, concentration-response data is currently being generated and made publicly available for 100s of toxicologically relevant endpoints in phase II of the Tox21 collaboration among the EPA, NCATS, the FDA and the NTP (Tice et al., 2013). Outcomes from these qHTS experiments can be used for numerous applications, including phenotypic screening (Kleinstreuer et al., 2014), genome-wide association mapping (Abdo et al., 2015) and prediction modeling (Eduati et al., 2015).

A qHTS assay produces one or more concentration-response curves for each tested compound. Here, we refer to a single concentration-response profile as a “repeat” (see section “Materials and Methods”). Each curve is typically evaluated using non-linear regression models. For example, the sigmoidal Hill model (Hill, 1910) is used to estimate the concentration at half-maximal response (*AC_50_*), a quantitative measure of chemical potency. Heteroscedastic responses and outliers should be taken into account using robust statistical modeling, such as the preliminary test estimation based methodology proposed by Lim et al. (2013). In addition to other characteristics of the concentration response curve, potency measures are important to determine how toxic or active a chemical is in the assay system. Estimates of compound potency or other response characteristics are extremely important for assessing toxicity in toxicology assessment or bioactivity in drug discovery applications. Recently, there has been considerable controversy in comparing two large-scale qHTS studies (Barretina et al., 2012; Garnett et al., 2012). Haibe-Kains et al. (2013) reported that the drug response data in these two studies were inconsistent with each other based on poor concordance of *IC_50_* and area under the curve (*AUC*) measures. This report and an accompanying commentary (Weinstein and Lorenzi, 2013) suggested that differences in laboratory protocols might account for this discordance and raised important questions about the validity and interpretation of current and future qHTS efforts. A number of studies have subsequently investigated the consistency of phamacogenomic drug response and investigated whether analytical assessments of consistency should take into account experimental features such as cell line (Cancer Cell Line Encyclopedia Consortium and Genomics of Drug Sensitivity in Cancer Consortium, 2015; Geeleher et al., 2016; Haverty et al., 2016; Safikhani et al., 2016a,d) and viability (Bouhaddou et al., 2016; Safikhani et al., 2016c), and suggested standardized assay methods and laboratory conditions (Mpindi et al., 2016; Safikhani et al., 2016b). Accounting for experimental factors during statistical analysis may help to improve the reliability and reproducibility of qHTS results (Ding et al., 2017). Nevertheless, such modeling approaches may require a prohibitively large number of repeated profiles for each chemical, and many experimental factors remain unknown or confounded in qHTS experiments.

Unfortunately, no systematic quality control (Q/C) procedure has yet been established for qHTS data. We believe that the lack of such a Q/C procedure may contribute to the ongoing debate surrounding the consistency of large-scale *in vitro* screening data. In this paper, we take a simple and principled Q/C approach to sort out chemicals with “inconsistent” response patterns so that the researcher may identify and avoid computing *AC_50_* values for potentially troublesome chemicals. Conversely, data with “consistent” responses across repeated profiles would produce *AC_50_* values that can be trusted and used for downstream analyses.

**FIGURE 1:**
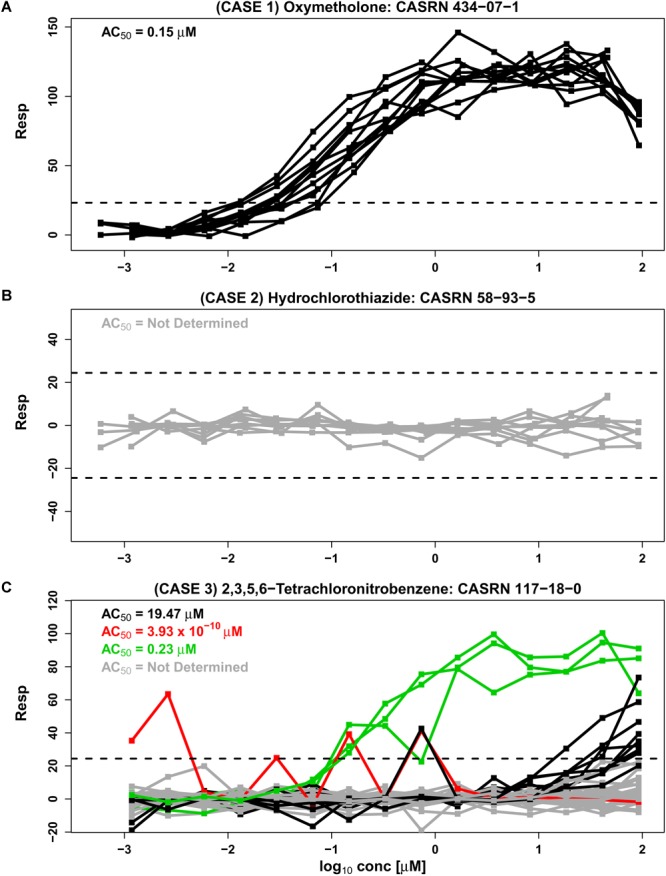
Three separate cases are represented by concentration-response data from the BG1 estrogen receptor agonist assay from phase II of the Tox21 collaboration (*tox21-er-luc-bg1-4e2-agonist-p2*). Responses are shown as a percentage of the assay positive control values after correction by DMSO negative controls (Inglese et al., 2006). The assay detection limits are indicated with dashed lines. An *AC_50_* value from the Hill model, calculated using the weighted average approach, summarizes the potency of each cluster (see section “Materials and Methods”). **(A)** Case 1 shows 12 similar response profiles from oxymetholone which extend beyond noise and group together into a single cluster. This case corresponds to two different supplier designations, two library preparation sites and two purities (A and D, representing “good” and “poor” purity, respectively) generated on six different experimental days. **(B)** Case 2 shows nine responses from hydrochlorothiazide which all lie within the noise band and correspond to three supplier sources, three library preparation sites, and a single purity (A) generated in six different experimental days. **(C)** Case 3 is represented by 42 response profiles from 2,3,5,6-tetrachloronitrobenzene corresponding to one supplier, three library preparation sites, one purity designation (A) and seven experimental days. A total of 29 of the 42 repeats lie within the noise band (shown in gray), and other profiles cluster by our proposed methodology *CASANOVA* described in this paper into the three disparate groups of 9, 3, and 1 repeats shown in black, green, and red, respectively. The separation of clusters in Case 3 is not explained by library preparation site or experimental day.

In the Tox21 initiative, multiple concentration-response curves are obtained for each compound tested in a qHTS study. However, this may not be the case with other qHTS studies, where only a single response curve is obtained for each tested compound. In some cases, the concentration-response patterns in Tox21 Phase II fall into a single cluster where response patterns are “similar” across all experimental repeats (e.g., Figure 1A,B, based on data from an estrogen receptor agonist assay). Concentration-response curves corresponding to oxymetholone in Figure 1A appear to be in a single cluster with all repeats exhibiting monotonic responses except at the highest concentration tested. Each curve crosses the upper noise bound (horizontal dashed line), suggesting that this compound is a candidate hit that may activate the estrogen receptor. Similarly, concentration-response data corresponding to hydrochlorothiazide in Figure 1B comprise one cluster pattern across all repeats since every concentration curve is within the noise limits, indicating that this chemical may not be active under the tested conditions. In examples such as Figure 1A, where all response curves are part of a single cluster, a Hill model (Hill, 1910; Shockley, 2015) or other appropriate non-linear model can be fit to the data in order to obtain potency estimates that summarize each curve. These individual potency estimates can then be used to obtain an overall potency estimate for the compound. Since the compound in Figure 1B appears to be inactive under the tested conditions, no potency estimate is obtained for this compound.

In the absence of systematic effects and artifacts, concentration-response curves for each chemical should be “similar” or within a single cluster across all experimental repeats of the compound (Hsieh et al., 2015). However, in Figure 1C the concentration-response patterns for 2,3,5,6-tetrachloronitrobenzene are split into four different clusters (indicated by different colors) across the experimental repeats. The *AC_5_*_0_ values for the three clusters with response values extending outside of the noise band range from 3.93 × 10^-10^ to 19.57 μM, representing a wide variance in potency associated with this compound. Unfortunately, the numerous examples of compounds with multiple response clusters could produce dramatically different potency estimates for the same compound. In such cases it can be very difficult to ascertain the correct concentration-response pattern for the tested compound, and its corresponding potency estimate, from the data alone. Chemical supplier, institutional site preparing the chemical library (e.g., NTP, FDA, and EPA), concentration-spacing, purity of the compound and other factors can systematically influence response trajectories (Tice et al., 2013). Such experimental factors are associated with different clusters in some instances. However, known design characteristics are not always associated with the observed response groupings.

An important purpose of qHTS assays is to estimate the potencies of active compounds for downstream analyses. In many cases, *AC_50_* values and point of departure values estimated from qHTS assays are used to discriminate between active and inactive compounds. Published studies incorporate *AC_50_* potency estimates derived from qHTS assays for predictive cheminformatics (Jamal et al., 2016), *in vivo* activity prediction modeling (Martin et al., 2011; Kleinstreuer et al., 2013; Anthony Tony Cox et al., 2016), screening for therapeutic leads (Martinez et al., 2016; Xu et al., 2016; Chen et al., 2017), drug sensitivity testing (Barretina et al., 2012; Garnett et al., 2012), *in vitro*-to-*in vivo* extrapolation (IVIVE) pharmacokinetic modeling (Rotroff et al., 2010; Wetmore et al., 2012), computational modeling of androgen receptor activity (Kleinstreuer et al., 2017), toxicity testing (Judson et al., 2016; Karmaus et al., 2016) and prioritization for targeted testing (Judson et al., 2010). It is crucial to identify and distinguish compounds that have single cluster response patterns across repeated runs from compounds with multiple cluster response patterns. Otherwise, the potency estimates derived from qHTS assays may not be reliable, as seen for 2,3,5,6-tetrachloronitrobenzene in Figure 1C where the potency estimates for different clusters are highly variable. Visual inspection of response profiles and manual curation of “flagged” compounds (Filer et al., 2017) are based on complex rule structures and do not address the quality control issue that is investigated here. Since 1000s of compounds are tested in each assay, there is a need for an automated quality control process to separate compounds with single cluster and multiple cluster response patterns before making activity calls and estimating the potency of biologically responsive agents. Here, we focus on the statistical identification of single cluster and multiple cluster compounds in a data driven framework, and do not address the separate problem of relating the data to pathways of interest (Hsieh et al., 2015).

## Materials and Methods

### Development of the *CASANOVA* Clustering Algorithm

A typical qHTS assay in Tox21 generates concentration-response data multiple times for each compound. Rather than referring to these multiple observations on each compound across concentrations as “replicates” we refer to them as “repeats.” In typical experimental designs “replication” refers to repeating the experiment several times under identical experimental conditions. This is not the case with qHTS studies. In qHTS, for a given compound the experiment is often repeated by varying suppliers, laboratories/agencies (sites) preparing the library, chemical purity, etc. In each instance a concentration curve is obtained and these concentrations curves cannot be viewed as conventional replicates.

We developed an automated clustering algorithm called *CASANOVA* to cluster intrachemical responses into single clusters using classical two-way analysis of variance (ANOVA). The workflow for *CASANOVA* is presented in Supplementary Figure 1. First, concentration-response repeats having all responses across the concentration range located entirely within the noise band are removed, where the noise band is defined as ± 3 standard deviations (σ) of the response at the lowest concentration tested in the experiment. qHTS studies typically base the assay detection limit on the variation in the DMSO negative controls (Hsieh et al., 2015), the DMSO controls and the lowest concentration (Huang et al., 2011), or the first two concentrations (Filer et al., 2017). Defining the detection limit based on just the DMSO negative controls could be problematic for antagonist assays in which the response at the lowest tested concentration relies on two different components: the DMSO controls and the agonist response needed to activate a nuclear hormone receptor. To be consistent across assay types and other studies in the literature, we chose to base the assay detection limit on the first tested concentration. In many, but not all, assays the variation in the DMSO negative control wells is very similar to the variation at the lowest tested concentration (Supplementary Figure 2).

Here, for each compound with at least two repeats extending beyond the assay detection limit of 3σ (or -3σ), an ANOVA model is fit to all *n* intrachemical response profiles. If all repeats within a compound lie within the noise band, the compound is designated “Case 2.” A grouping factor to divide the concentration space is essential to our approach. In this study, we focus on the 15-point concentration response profiles generated in phase II of Tox21 and use five “3-concentration” bins to define a five-level “concentration” grouping factor termed *CONC*. We consider each concentration-response profile in the experiment to be a “repeat,” and *REPEAT* is used as a second factor in the model. Response *R_ijk_* for concentration bin *i* (*CONC_i_*), repeat *j* (*REPEAT_j_*), and an interaction term (γ_ij_) for observation *k* is modeled using the compound-specific ANOVA model

(1) $$\begin{array}{c} R_{ijk}=\mu +CONC_{i}+REPEAT_{j}{+\gamma }_{ij}{+\varepsilon }_{ij} \end{array}$$

where μ is the overall mean and *𝜖_ijk_* represents random error for concentration bin *i*, repeat *j* and observation *k*. The γ term is first tested for statistical significance within each compound. If the interaction term is significant at the user specified level of α (H_0_: γ_11_ = γ_12_ = … = γ_nn_), then the *REPEAT* term is tested for significance at the α level (H_0_: *REPEAT_1_* = … = *REPEAT_n_*). Unless otherwise noted, we used α = 0.05 for all analyses presented here. If *REPEAT* is also significant, then repeats are ranked by mean response averaged over all levels of the *CONC* factor and significant pairwise differences between neighboring repeats in the ranked list are used to group repeats into distinct clusters. Subgroup analysis then proceeds by ranking mean response values within the highest *CONC* bin. Significant pairwise differences between neighboring repeats in the ranked list within this bin are used to further divide these clusters into new subclusters. The subgroup analysis proceeds for each *CONC* bin level (from the highest concentration to lowest concentration). If γ is significant, but *REPEAT* is not significant, only the subgroup analysis is performed. If the γ term is not significant, but *REPEAT* is significant, repeats are ranked by mean response averaged over all levels of the *CONC* factor and significant pairwise differences between neighboring repeats in the ranked list are used to group repeats into distinct clusters.

Once the clusters of similar dose profiles have been determined, the mean response values lying above (or below) the noise band across all concentration bins are compared with the upper (or lower) detection limit using the one sample *t*-test (α = 0.05) in order to distinguish between “conclusive” clusters that are statistically separated from the noise band and “inconclusive” clusters that are not statistically different from the noise band detection limit. “Case 1” compounds are composed of *n* single cluster repeats, where *n* refers to all the tested repeats within a compound. “Case 3” compounds each contain multiple cluster response patterns, where one of the clusters can potentially be repeats with all responses located entirely within the noise band. Supplementary Figure 3 describes the five different classes of possible compound classification outcomes.

### Description of Tox21 Phase II Data Sets

Publicly available Tox21 Phase II data was obtained from https://tripod.nih.gov/tox/. This qHTS data involves approximately 10,000 compounds screened for activity related to stress response, nuclear hormone receptor activity, or cell viability. The nuclear receptor hormone assays were performed in agonist and antagonist (or inhibitor) modes and are used to investigate activation or inhibition activities of the given assay. Multiple channel readouts for beta-lactamase gene reporter assays consisted of ch1, ch2 and ratio (ch2/ch1) data, and in those cases we used the ratio data to represent the assay signal. A total of 15 concentrations were evaluated with concentrations typically ranging from approximately 5 × 10^-4^ μM to about 100 μM (Tice et al., 2013). As part of phase II of Tox21, the library is screened three times with compounds located in different well positions during each experimental run (Tice et al., 2013). The raw plate reads were normalized using the positive and negative control wells and subsequently corrected for row, column, and plate effects using linear interpolation (Inglese et al., 2006). A total of 43 of the 47 publicly available bioassay data sets represented by 72 different readouts from phase II of the Tox21 collaboration were selected for analysis in this study due to their comparable experimental design of 15-point concentration response data generated in triplicate runs. We dropped 4 of the 47 publicly available data sets from our analysis because their study design was not directly comparable with the other 43 data sets; 2 of the assays were conducted as 4- or 8-point concentration-response study designs and 2 additional assays were unreplicated time course experiments.

*AC_50_* values, and corresponding standard errors (*SE*), of individual concentration-response curves were estimated from the data using the Hill model after removing outliers as described previously (Shockley, 2012). The *AC_50_* from each cluster in a single compound was estimated with a weighted approach using (1/*SE*)^2^ as weights and the *weighted.mean()* function in R.

### Simulation Studies to Evaluate the *CASANOVA* Algorithm

The performance of *CASANOVA* to correctly cluster similar patterns and separate disjoint patterns, was evaluated in simulation studies conducted across a range of assay noise levels chosen to resemble the characteristics found in Tox21 Phase II qHTS data. A total of 2,000 simulated compounds with at least one response outside of the noise band were generated from either the Hill model (sigmoidal curves) or the gain-loss model (“bell-like” curves) (Shockley, 2016; Filer et al., 2017). The parameters of the simulation study were based on observed data in the Tox21 Phase II data sets. Of the 43 publicly available Tox21 data sets (with 72 readouts) examined here, we chose four assay readouts that span the range of assay noise based on negative control DMSO plates (see Supplementary Figure 4) and the lowest tested concentration levels (Supplementary Figure 5). These selected readouts come from assays with low noise (data set 1: *tox21-elg1-luc-agonist*), moderate-low noise (data set 2: *tox21-are-bla-p1*), moderate-high noise (data set 3: *tox21-er-luc-bg1-4e2-agonist-p2*), and high noise (data set 4: *tox21-fxr-bla-agonist-p2*). The proportion of chemicals with *N* suppliers (*N = 1, 2, 3, 4* in the Tox21 Phase II experiments) in each of the selected data sets was calculated (see Supplementary Table 1) and used as input probabilities for simulating the number of clusters per compound. Similarly, the proportion of compounds with *N* repeats per supplier (*N* = 3, 6, 9, 12, 42, 45, 48, 51, 54) was determined empirically for the four selected datasets (see Supplementary Table 2) and used as input probabilities for simulating the number of repeats per cluster in each compound. An ANOVA model in Eq. (1) was fit to compounds containing at least two repeats with detectable responses as described above. For each chemical, the ANOVA mean squared error (*MSE*), the range defined by maximum observed response – minimum observed response (*ResponseRange*) and the coefficient of variation (*CV*) defined by $\sqrt{MSE}$/ResponseRange was calculated. These values, presented in Supplementary Table 3, were used to similate the data as described in greater detail below.

Simulated concentration-response curves are randomly chosen for each cluster based on a three-parameter Hill equation model or a four-parameter “gain-loss” model. The three-parameter Hill model is described by:

(2) $$\begin{array}{c} E(R_{ij})=\frac{RMAX_{j}}{1+10^{\left\{-h_{j}[log_{10}C_{i}-log_{10}AC_{50,j}]\right\}}} \end{array}$$

where *R_ij_* is a normalized response (% of positive control activity) for the *jth* repeat, *RMAX_j_* represents maximal response, *h_j_* is the slope parameter, *C_i_* is the compound concentration, and *AC_50,j_* is the concentration for half-maximal activity. Similar to a previous study (Shockley, 2016), the concentrations are based on equivalent log_10_ concentration spacing from 0.0001 to 100 μM in 15-point concentration-response curves. The “gain-loss” model is given by

(3) $$\begin{array}{c} E(R_{ij})=RMAX_{j}(\frac{1}{1+10^{\left\{h_{j}(log_{10}AC_{50(G),j}-log_{10}C_{i})\right\}}})\times (\frac{1}{1+10^{\left\{h_{j}(log_{10}C_{i}-log_{10}AC_{50(L),j})\right\}}}) \end{array}$$

where *RMAX_j_* is the shared upper asymptote, both bottom asymptotes are set to zero, *h_j_* is the slope parameter, *AC_50(G),j_* is the concentration of half-maximal response in the gain direction and *AC_50(L),j_* is the concentration of half-maximal response in the loss direction (Filer et al., 2017).

For each cluster, the mean *RMAX_j_* value (μ_RMAX_) is selected using random deviates from the uniform distribution on (3σ, *ResponseRange*) and *RMAX_j_* is drawn from *N*(μ_RMAX_, *MSE*). The slope parameter *h_j_* is drawn from |N(1,9)|. For each cluster, mean values (*MEAN*) of *log_10_AC_50,j_* from the Hill model, or *log_10_AC_50(G),j_* from the “gain-loss” model, are randomly selected from (0.0001, 0.001, 0.01, 0.1, 1, 10, 100), or from (0.0001, 0.01, 1, 100) with equal probabilities and without replacement, across clusters for 10-fold *AC_50_* spacing and 100-fold *AC_50_* spacing, respectively. Mean values of *log_10_AC_50(G),j_* are randomly selected from (0.0001, 0.001, 0.01, 0.1, 1, 10, 100) with equal probabilities where *log_10_AC_50(L),j_* – *log_10_AC_50(G),j_* ≥ 1 for 10-fold *AC_50_* spacing, or from (0.0001, 0.01, 1, 100) with equal probabilities where *log_10_AC_50(L),j_* – *log_10_AC_50(G),j_* ≥ 2 for 100-fold *AC_50_* spacing. If no *log_10_AC_50(L),j_* values within the selected range meet this criterion, *log_10_AC_50(L),j_* is set to 1000. The random realization of the mean *log_10_AC_50,j_* value, or *log_10_AC_50(G),j_*, is drawn from *N*(MEAN, σ), where σ = 1/6 is selected so that ∼99.7% of all *AC_50_* values between clusters are separated at least 10- or 100-fold, depending on the simulation scenario. After determining the parameters for each cluster, response data was simulated by adding heteroscedastic noise to ideal curves with *N*(0, *R_ij_* × *CV*), where *R_ij_* is given from Eq. (2) or Eq. (3) above. Summary statistics for the simulated data are given in Supplementary Tables 4, 5.

## Results

### Applying *CASANOVA* to Tox21 Phase II Data

*CASANOVA* was applied to publicly available Tox21 Phase II data related to stress response, nuclear receptor signaling and cell viability in order to assess the consistency of intra-chemical response patterns within and between assays. We selected 43 of the 47 publicly available data sets since these data sets were generated using a similar experimental design (i.e., 15-point concentration-response data generated in three experimental runs). These 43 data sets correspond to 72 different readouts, where many of the agonist and antagonist assays monitored cytotoxicity as well as the response in the specified assay mode. A total of 7,229 chemicals were represented in all 72 readouts.

**FIGURE 2:**
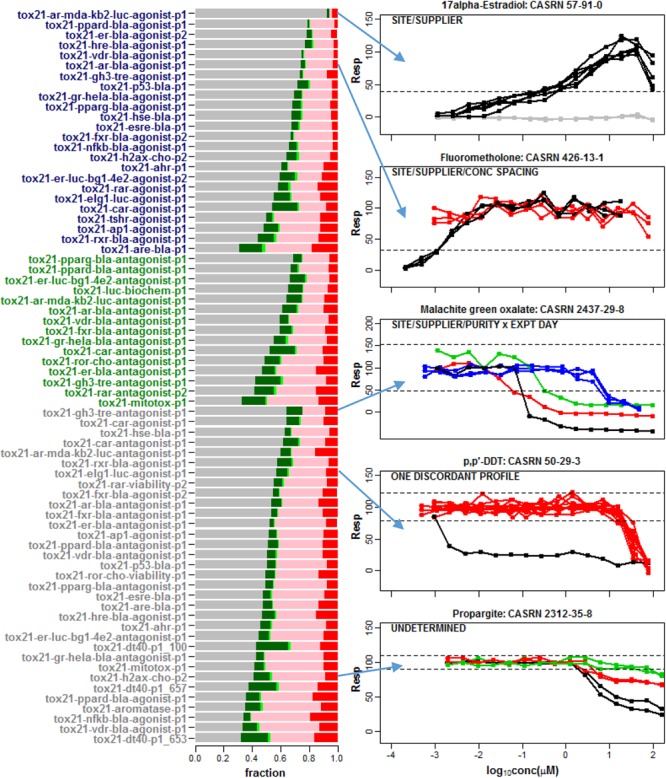
A barplot was used to summarize the response patterns corresponding to 72 assay readouts from 43 different data sets. A total of 7,229 chemicals were common among all 43 data sets. In the barplot, the gray regions correspond to the fraction of chemicals clustered in the noise band (Case 2), the dark green regions refer to a single detectable cluster well-separated from the noise band (Conclusive Case 1), the light green regions represent a single cluster with response points not statistically separable from noise (Inconclusive Case 1), the pink regions correspond to multiple clusters with response points not statistically separable from the noise band (Inconclusive Case 3) and the red regions refer to multiple clusters well-separated from the noise band (Conclusive Case 3). Agonist assay labels are shown in dark blue, antagonist/inhibitor assay labels are shown in green and viability assay labels are shown in gray. Selected compound profiles from assays with multiple clusters (Conclusive Case 3) are shown to the right of the barplot. Known factors associated with different clusters are indicated in the upper left of each plot. These factors include supplier, library preparation site, concentration spacing, compound purity and experimental day. None of these factors explain the different patterns observed in the last two plots. Hence, adjusting or normalizing the concentration-response data for these known factors will not necessarily eliminate multiple cluster response patterns among repeats within a compound in qHTS data.

The barplot in Figure 2 shows the fraction of these compounds that were classified as single clusters that are well-separated from the noise band (Conclusive Case 1), single clusters that extend outside of the noise band and points outside the noise threshold are not significantly different from the noise band (Inconclusive Case 1), non-responsive with all repeats located within the noise band (Case 2), multiple clusters where at least one cluster extends outside the noise band and points outside the noise threshold are not significantly different from the noise band (Inconclusive Case 3) or multiple clusters for which at least one cluster extends significantly beyond the noise band (Conclusive Case 3). Most chemicals do not exhibit any response in the tested assay conditions (Case 2). The fraction of single clusters among all 7,229 compounds with at least one detectable response in an assay ranges from 1.6% (*tox21-vdr-agonist-p1*) to 23.8% (*tox21-dt40-p1_100*) across the 72 readouts. As shown in the plots for selected compounds in Figure 2, this multiplicity in response is sometimes associated with one or more known experimental design factors such as supplier, library preparation site, compound purity, concentration spacing, or experimental day (Figure 2). For example, in the top panel of Figure 2 supplier is confounded with site of library preparation so that one or both of these two experimental factors can potentially account for the separation of response patterns into two different clusters.

**FIGURE 3:**
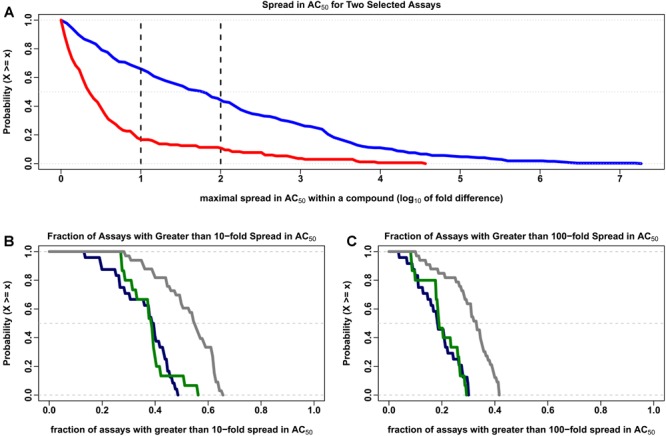
Complementary empirical cumulative distribution (CCDF) describing the variability in *AC_50_* values. The maximal range of *AC_50_* values (on the log_10_ scale) was calculated for each compound in which two or more clusters were identified outside of the noise region for each of the 7,729 compounds investigated in the 43 data sets described in the text. The order of magnitude differences in intrachemical potency estimates shown here represent only those cases in which the calculated *AC_50_* is between 10^-5^ and 1000 μM, which covers the typical testing concentration range of ∼10^-4^ to 100 μM evaluated in these assays. The number of compounds meeting this criterion ranged from 42 to 774 in the 72 assay types evaluated here, with a median of 255 compounds. **(A)** The CCDF (or 1-CDF) plots describing the proportion of compounds (y-axis) for a given spread in *AC_50_* (x-axis) in the *tox21-er-luc-bg1-4e2-antagonist-p1* viability assay (blue) and the *tox21-gh3-tre-agonist-p1* agonist assay (red) are displayed. The vertical black lines indicate 10- and 100-fold differences in the calculated range of *AC_50_* values. **(B)** The CCDF for the fraction of the 72 assays with greater than 10-fold range in *AC_50_* values (y-axis) for a given spread in *AC_50_* (x-axis) are shown for the agonist (dark blue), antagonist/inhibitor (dark green) and viability (dark gray) assays. **(C)** The CCDF for the fraction of the 72 assays with greater than 100-fold range in *AC_50_* are shown for the same agonist, antagonist/inhibitor and viability assays presented in **(B)**.

The Hill model (Hill, 1910) was used to estimate the concentration for half maximal activity (*AC_50_*) for the 7,229 compounds common to all 43 data sets. Compounds with two or more clusters outside of the noise band and estimated *AC_50_* values within about 10-fold of the typical concentration range in the assays (10^-5^ to 1,000 μM) were evaluated further in order to discover the variability in *AC_50_* estimates within a multiple cluster compound. In Figure 3A, the percentage of multi-cluster compounds with *AC_50_* estimates greater than 10-fold ranged from 16.7% for *the tox21-gh3-tre-agonist-p1* agonist assay to 65.6% for the *tox21-er-luc-bg1-4e2-antagonist-p1* viability assay. The percentage of multi-cluster compounds with *AC_50_* estimate differences greater than 100-fold ranged between 10.7 and 43.8% for these two assays, respectively. The fraction of compounds with multiple cluster responses was not statistically different between agonist and antagonist/inhibitor assays. However, the distribution of multiple cluster compounds was greater in viability assays compared to the agonist and antagonist/inhibitor assays, when considering 10-fold (Figure 3B) or 100-fold (Figure 3C) potency differences (*p* < 0.001 using the two-sided Kolmogorov–Smirnov test). In Figure 3B, about 38% of the 7,729 tested compounds have at least a 10-fold spread in *AC_50_* estimates in half of the agonist and antagonist/inhibitor assays, whereas about 54% of the tested compounds have at least a 10-fold spread in *AC_50_* estimates in half of the viability assays. In Figure 3C, about 18% of the tested compounds have at least a 100-fold spread in *AC_50_* estimates in half of the agonist and antagonist/inhibitor assays, while about 32% of the tested compounds have at least a 100-fold spread in *AC_50_* estimates in half of the viability assays.

### Simulation Studies to Evaluate the Performance of *CASANOVA*

Simulation error rates were determined by averaging error rates from each simulated data set of 2,000 compounds across 100 different simulated runs. Error rates were calculated for each run based on the proportion of compounds with a given error type. “Type A” error was assigned to a compound when the *CASANOVA* approach incorrectly separated any two repeats from a true cluster (i.e., when a true single cluster compound was classified as a Conclusive Case 3). Conversely, a “Type B” error was assigned to a compound when any two repeats from separate clusters were falsely combined (i.e., when a true multiple-cluster compound was classified as a Conclusive Case 1). In both cases, these error rates are less than 5% with *p* < 0.05 and *p* < 0.10 as the selected criterion for identifying and separating clusters for either 10-fold *AC_50_* spacing or 100-fold *AC_50_* spacing (Table 1).

**Table 1:** *CASANOVA* classification errors for the given *p*-value threshold.

	*p*-Value threshold							
	0.00	0.001	0.01	0.05	0.10	0.20	0.50	1.00
**10-fold *AC_50_* spacing**					
Dataset 1^a^								
errorA	0.019	0.019	0.019	0.023	0.031	0.051	0.151	0.585
errorB	0.418	0.102	0.057	0.032	0.022	0.014	0.001	0.000
Dataset 2^b^								
errorA	0.022	0.022	0.023	0.027	0.036	0.058	0.163	0.565
errorB	0.392	0.104	0.059	0.033	0.022	0.014	0.006	0.000
Dataset 3^c^								
errorA	0.017	0.017	0.018	0.022	0.028	0.047	0.141	0.588
errorB	0.423	0.098	0.055	0.030	0.021	0.013	0.005	0.000
Dataset 4^d^								
errorA	0.031	0.031	0.032	0.037	0.047	0.072	0.182	0.416
errorB	0.312	0.109	0.064	0.035	0.024	0.014	0.004	0.000
**100-fold *AC_50_* spacing**					
Dataset 1^a^								
errorA	0.014	0.014	0.015	0.020	0.028	0.050	0.166	0.606
errorB	0.348	0.037	0.014	0.005	0.003	0.001	0.000	0.000
Dataset 2^b^								
errorA	0.017	0.017	0.018	0.024	0.033	0.058	0.178	0.583
errorB	0.331	0.039	0.015	0.005	0.003	0.002	0.001	0.000
Dataset 3^c^								
errorA	0.013	0.013	0.014	0.018	0.025	0.046	0.154	0.609
errorB	0.351	0.034	0.012	0.004	0.002	0.001	0.000	0.000
Dataset 4^d^								
errorA	0.025	0.025	0.026	0.034	0.045	0.074	0.202	0.521
errorB	0.273	0.048	0.019	0.006	0.003	0.001	0.000	0.000

*^*a*^*tox21-elg1-luc-agonist*, ^*b*^*tox21-are-bla-p1*, ^*c*^*tox21-er-luc-bg1-4e2-agonist-p2*, ^*d*^tox21-fxr-bla-agonist-p2.*

### Simulation Studies to Evaluate AC_50_ Parameter Estimation

The bias and precision (1/variance) of *AC_50_* estimation was evaluated in a separate simulation study. This simulation reflects the situation in which potency is estimated for single cluster compounds (Case 1). A total of 2,000 chemicals were simulated in activation mode, with increasing responses for increasing concentrations across a range of concentrations between 0.1 nM and 100 μM, where *AC_50_* values were set to 0.001 μM (upper asymptote only), 0.1 μM (both asymptotes) or 10 μM (lower asymptote only). *RMAX* was considered at three values (25, 50, and 100% of positive control). The hill parameter was set to 1 for all curves in this simulation. Residual errors were modeled as *ERROR* ∼*N*(0, σ^2^) with σ = 5% or 10%.

Outliers were removed (Wang et al., 2010), separate curves were fit to each response curve and the *log_10_AC_50_* parameter value was calculated for each profile (Shockley, 2012). We evaluated *n* profiles per compound for *n* = 3, 6, 9, or 12. For each compound, profile-specific estimates were summarized using the average, median or weighted average of the estimates, or a single model fit (Shockley, 2015). As described above, the weighted average approach uses (1/*SE*)^2^ for weights, where *SE* is the standard error of the parameter estimate. The bias was less than 0.01 (1.02-fold) and the variance was less than 0.04 (1.1-fold) when both plateaus/asymptotes were present in the simulated sigmoidal curve for σ = 5% (Table 2). These errors were larger for σ = 10% (Supplementary Table 6). The weighted average approach produced the most repeatable results, where both bias and variance of the estimated *log_10_AC_50_* for a compound were typically within one order of magnitude (10-fold).

**Table 2:** Bias and variance of *log_10_AC_50_* parameter for Hill model curves (5% error).

True	True		Bias (and variance) of *log_10_ AC_50_*			
*AC_50_*	*RMAX*	*n*	Avg	Median	WT Avg	One model
1.00e-03 Upper plateau only	25	3	1.26 (4.07)	0.42 (2.63)	0.03 (0.61)	0.52 (4.07)
	25	6	1.22 (2.13)	0.21 (0.61)	0.10 (0.07)	0.44 (3.16)
	25	9	1.19 (1.42)	0.20 (0.08)	0.11 (0.04)	0.46 (3.06)
	25	12	1.24 (1.10)	0.08 (0.10)	0.10 (0.03)	0.41 (2.84)
	50	3	0.28 (0.52)	0.07 (0.19)	0.05 (0.10)	0.05 (0.11)
	50	6	0.27 (0.24)	0.03 (0.02)	0.08 (0.02)	0.04 (0.05)
	50	9	0.26 (0.14)	0.02 (0.01)	0.09 (0.01)	0.04 (0.06)
	50	12	0.26 (0.11)	0.02 (0.01)	0.09 (0.01)	0.04 (0.06)
	100	3	0.02 (0.01)	0.01 (0.01)	0.03 (0.01)	0.01 (0.01)
	100	6	0.03 (*)	0.01 (*)	0.03 (*)	0.01 (0.01)
	100	9	0.03 (*)	0.01 (*)	0.04 (*)	0.01 (0.01)
	100	12	0.03 (*)	* (*)	0.04 (*)	0.01 (0.01)
0.1 Upper and lower plateaus	25	3	0.13 (1.52)	0.01 (0.05)	* (0.04)	0.01 (0.03)
	25	6	0.08 (0.63)	0.01 (0.02)	* (0.02)	* (0.03)
	25	9	0.09 (0.45)	* (0.01)	* (0.01)	* (0.03)
	25	12	0.07 (0.32)	0.01 (0.01)	* (0.01)	0.01 (0.03)
	50	3	* (0.01)	* (0.01)	* (0.01)	* (0.01)
	50	6	* (*)	* (*)	* (*)	* (0.01)
	50	9	* (*)	* (*)	* (*)	* (0.01)
	50	12	* (*)	* (*)	* (*)	* (0.01)
	100	3	* (*)	* (*)	* (*)	* (*)
	100	6	* (*)	* (*)	* (*)	* (*)
	100	9	* (*)	* (*)	* (*)	* (*)
	100	12	* (*)	* (*)	* (*)	* (*)
10 Lower plateau only	25	3	1.86 (4.78)	1.04 (5.08)	0.13 (1.65)	0.72 (4.72)
	25	6	1.91 (2.42)	0.72 (1.82)	0.06 (0.43)	0.73 (5.10)
	25	9	1.90 (1.70)	0.48 (1.20)	0.11 (0.11)	0.78 (5.27)
	25	12	1.90 (1.21)	0.37 (0.56)	0.09 (0.10)	0.75 (4.93)
	50	3	0.74 (1.08)	0.30 (0.78)	0.03 (0.34)	0.09 (0.15)
	50	6	0.74 (0.51)	0.19 (0.16)	0.03 (0.13)	0.12 (0.30)
	50	9	0.77 (0.36)	0.14 (0.06)	0.04 (0.07)	0.13 (0.27)
	50	12	0.75 (0.27)	0.12 (0.03)	0.07 (0.03)	0.12 (0.24)
	100	3	0.14 (0.10)	0.06 (0.03)	0.01 (0.04)	0.02 (0.01)
	100	6	0.14 (0.06)	0.04 (0.01)	0.02 (0.02)	0.02 (0.01)
	100	9	0.14 (0.04)	0.03 (0.01)	0.03 (0.01)	0.02 (0.01)
	100	12	0.14 (0.02)	0.03 (*)	0.03 (*)	0.02 (0.01)

*Values of bias or variance less than 0.01 are indicated by “^∗^”. ^∗^(^∗^) indicates that both the bias and the variance are less than 0.01.*

## Discussion

Millions of dollars are being invested in developing qHTS assays and there are far reaching economic and public health implications for these large-scale studies. We believe that there is a pressing need for a rigorous, yet simple, Q/C process such as the one we offer in this work. Chemical genomics efforts inevitably involve multiple sources of variation imposed by limited resources and the technological constraints of robotic plate handling (Attene-Ramos et al., 2013). On the one hand, it can be advantageous to have compound activity data generated across multiple design factors in order to increase the chances that an observed response is related to the biological assay of interest rather than technical error (Ding et al., 2017). However, differences in chemical supplier, compound purity, laboratory protocol, or the day of the experiment may produce systematic errors that vary from chemical to chemical. Assay interference arising from autofluorescence and compound-induced cytotoxicity can also cause misleading signals (Tice et al., 2013; Hsieh et al., 2015). Other influential factors may be unknown or difficult to take into account (Malo et al., 2006). The proximity of wells in microtiter test plates may yield misleading signals due to signal flare or inadvertent contamination. Well-composition could also change over time due to evaporation, alterations in dissolvability, volatility, or chemical reaction. Artifacts can have an unpredictable effect on the biological response (Hsieh et al., 2015). Unfortunately, these design restrictions may lead to discordant intrachemical response patterns even after data normalization.

In this article we present a simple methodology to group intrachemical repeats in an automated manner. In theory, if a compound is active, then we expect the responses to be active at the lowest tested concentration (i.e., exceeding the noise limits), monotonic, or partially ordered (e.g., up-turn or down-turn responses) with concentrations. Our data driven approach to cluster compound-specific response patterns, termed *CASANOVA*, finds clusters in which repeats group together across the entire concentration-response domain as well as clusters which distinguish repeats in concentration subgroups.

We assessed the consistency of intra-chemical response patterns within and between Tox21 Phase II assays interrogating nuclear receptor activity and stress response. While most chemicals do not exhibit any response in the tested assay conditions, a fraction of compounds (i.e., 1.6 to 23.8% across the tested assays) with at least one profile extending outside of the noise band represent single cluster response patterns (Figure 2). Multiplicity in response can often be attributed to one or more known experimental design factors. Still, it may not be possible to account for all confounding factors associated with an observed disparity of responses (e.g., Figure 1C). The wide range of *AC_50_* estimates obtained for the same compound in experimental data sets (Figure 3) underscores the importance of a clustering algorithm such as *CASANOVA* to identify compounds with single cluster patterns of response. Otherwise, compound potency estimates may not be reliable.

Simulation studies were used to evaluate the ability of *CASANOVA* to cluster compound profiles into reliable subgroups and provide suitable *AC_50_* potency estimates. The overall error rates for *CASANOVA* to correctly cluster similar patterns (“Type A” errors) and separate disjoint patterns (“Type B” errors) was found to be less than 5% across a range of simulation studies based on Tox21 Phase II qHTS data using 10- or 100-fold *AC_50_* spacing. We employed a *p*-value threshold of 0.05 to describe patterns in the Tox21 Phase II data. However, the results from our simulation studies reveal that selecting a less stringent *p*-value threshold (e.g., *p* < 0.10) can be used to increase the “Type A” error and decrease the “Type B” error according to different research motivations. Assuming that all the profiles belong to a single cluster, simple averaging of individual *AC_50_* estimates leads to the greatest bias and least precise estimates. However, the weighted average approach produces the most repeatable results, where both bias and variance are generally within one order of magnitude.

The *CASANOVA* approach provides an unsupervised method to agnostically separate multiple cluster response compounds from compounds with reasonably concordant concentration-response repeats. Our approach therefore avoids a complicated modeling effort to account for all potentially influential variables in the data, many of which may not be explicit or identifiable in any given study. Compound potency estimates in qHTS experiments can vary substantially (well over 100-fold in some cases) in large scale *in vitro* bioassay data due to multiple cluster intrachemical responses. Lim et al. (2013) discussed possible strategies to derive optimal experimental designs for qHTS experiments to improve the precision of potency estimates and statistical inference on these parameters. Nevertheless, *CASANOVA* can improve the detection of single cluster intrachemical repeats and potency estimation for candidate hits irrespective of the underlying study design. Multiple cluster compounds identified using *CASANOVA* can be studied further to understand the source of the variation which may arise from technological disturbances such as compound carryover, interference between signal channels, autofluorescence, or potential fluctuations in the laboratory environment. However, by focusing research efforts on compounds with single cluster response patterns, potency estimation is expected to be more accurate and precise. We anticipate that *CASANOVA* can be applied to other types of sequential data types involving non-linear responses, including dose-response and longitudinal genomics studies, where divergent responses in subregions of the data are important. The R code for CASANOVA is available upon request or can be downloaded online from www. niehs.nih.gov/research/atniehs/labs/bb/staff/shockley/index.cfm.

## Author Contributions

KS and SP designed the study, analyzed the data, and wrote the manuscript. KS, SP, SH, SG, and SL edited the manuscript. SP, SG, and SL conceived the application of two-way ANOVA algorithm for qHTS. SH performed automation of *CASANOVA*.

## Conflict of Interest Statement

The authors declare that the research was conducted in the absence of any commercial or financial relationships that could be construed as a potential conflict of interest.
