# Supplementary material for: Quality Control of Quantitative High Throughput Screening Data
Source: Front Genet. 2019 May 9;10:387. doi: 10.3389/fgene.2019.00387 (PMC6520559; doi:10.3389/fgene.2019.00387)
Supplement: Supplementary file 1 [file Data_Sheet_1.pdf]

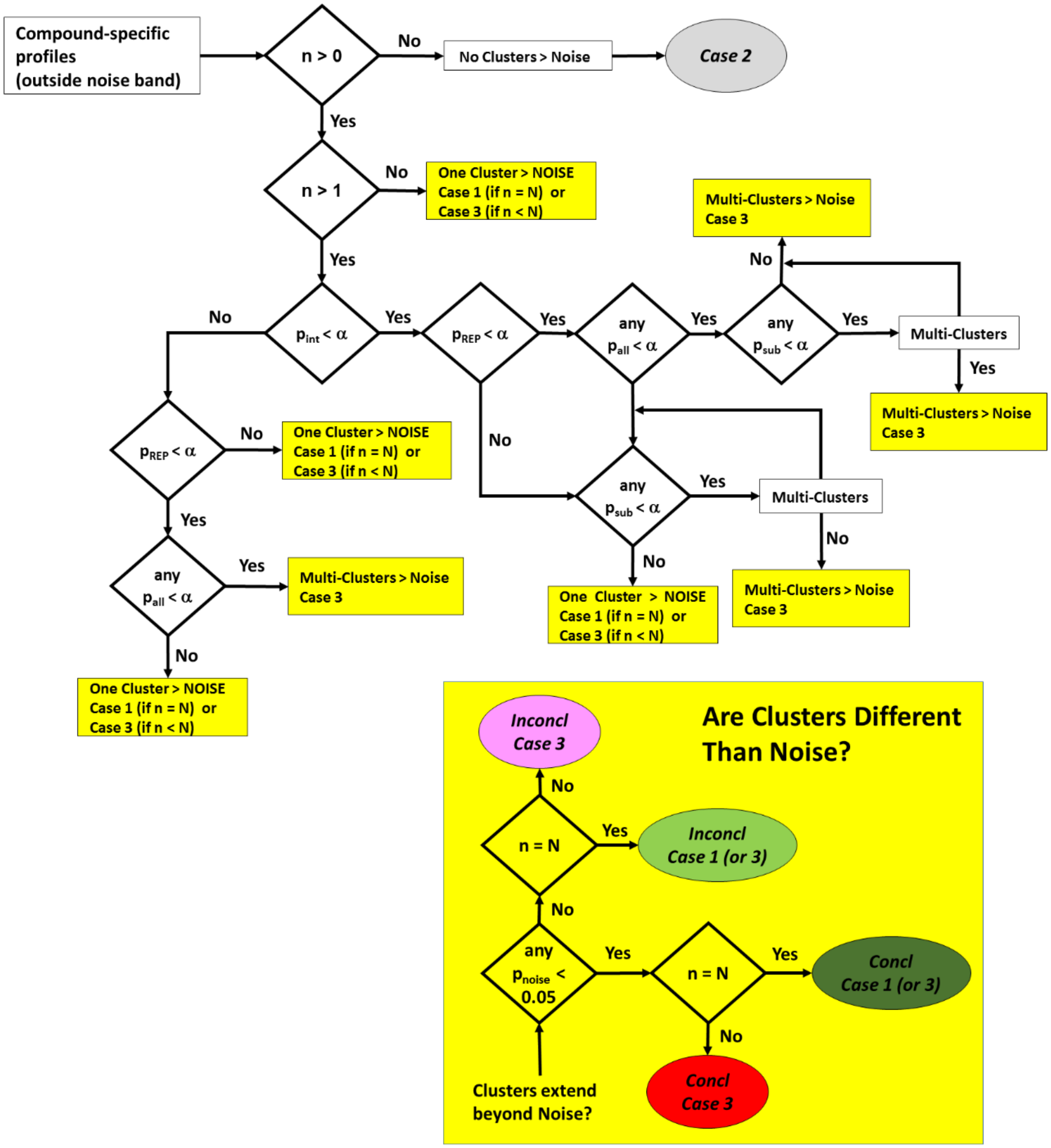

Supplementary Figure 1. The *CASANOVA* algorithm. Within each compound of *N* repeats, the repeats with at least one response outside the noise band (*n*, where $n \leq N$) are sorted into different clusters. If all repeats are within the noise band (*n*=0), the compound is assigned "Case 2". Concentration-response data for the compound are fit to a two-way ANOVA model with factors corresponding to the user-specified concentration bin, repeat, and repeat x concentration bin interaction as described in the **Methods**. The significance of the repeat (p_{REP}) and interaction terms (p_{int}) is computed using an F-test. When $p_{REP} < \alpha$, repeats are ordered by mean response across all concentration bins and pairwise contrasts between adjacent means are used to define clusters ($p_{all} < \alpha$). When $p_{int} < \alpha$, repeat responses are subset by concentration group (from highest to lowest) and pairwise contrasts between adjacent means are used to define subclusters ($p_{sub} < \alpha$). For each cluster with repeats that extend outside of the noise band (yellow boxes), one-sample t-tests are used to test whether all responses outside the noise band across concentration groups are significantly greater than (or less than) noise ($p_{noise} < 0.05$ for responses > noise or $p_{noise} < 0.05$ for responses < noise). Clusters are designated "conclusive" (different from the noise band) or "inconclusive" (not statistically different from the noise band) based on the separation of the response values from noise. The final clusters may contain a cluster with all the profiles located within the detection band (i.e., for n < N). "Case 1" compounds are composed of one cluster of *n* repeats ($n = N$). "Case 3" compounds contain multiple clusters (where one cluster can be repeats entirely within noise).

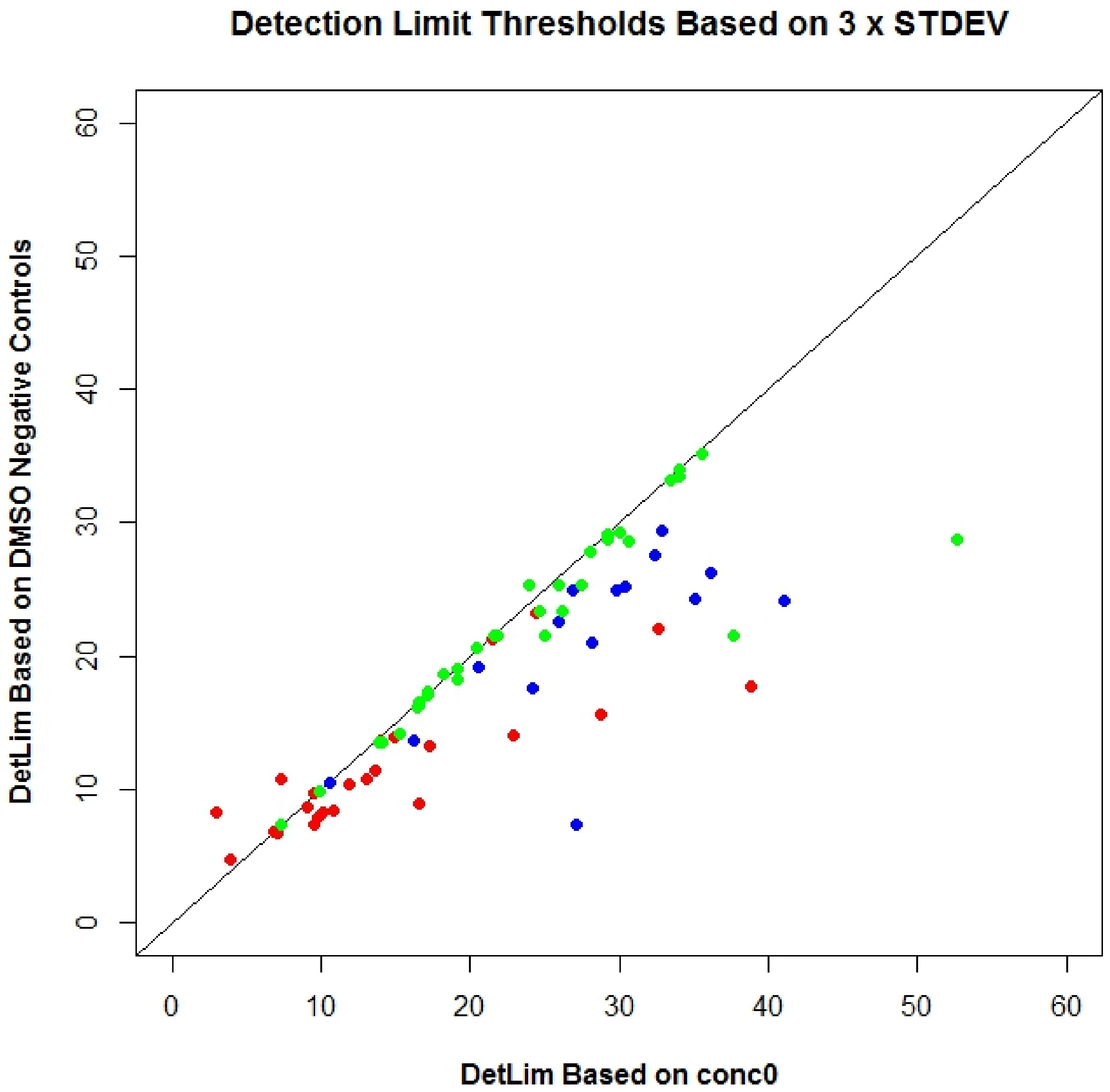

Supplementary Figure 2. Comparing the assay detection limit approaches based on the lowest tested concentration (*conc0*) and the DMSO negative control wells. The red dots represent the agonist assay readouts (ratio readout for beta-lactamase assays or the luciferase channel readout), the blue dots represent the antagonist assay readouts and the green dots represent the viability assays. The results are presented at the 0% baseline for all readouts. For antagonist/inhibitor assays and viability assays, the 100% baseline is adjusted to the zero baseline by subtracting 100% from the 100% baseline values.

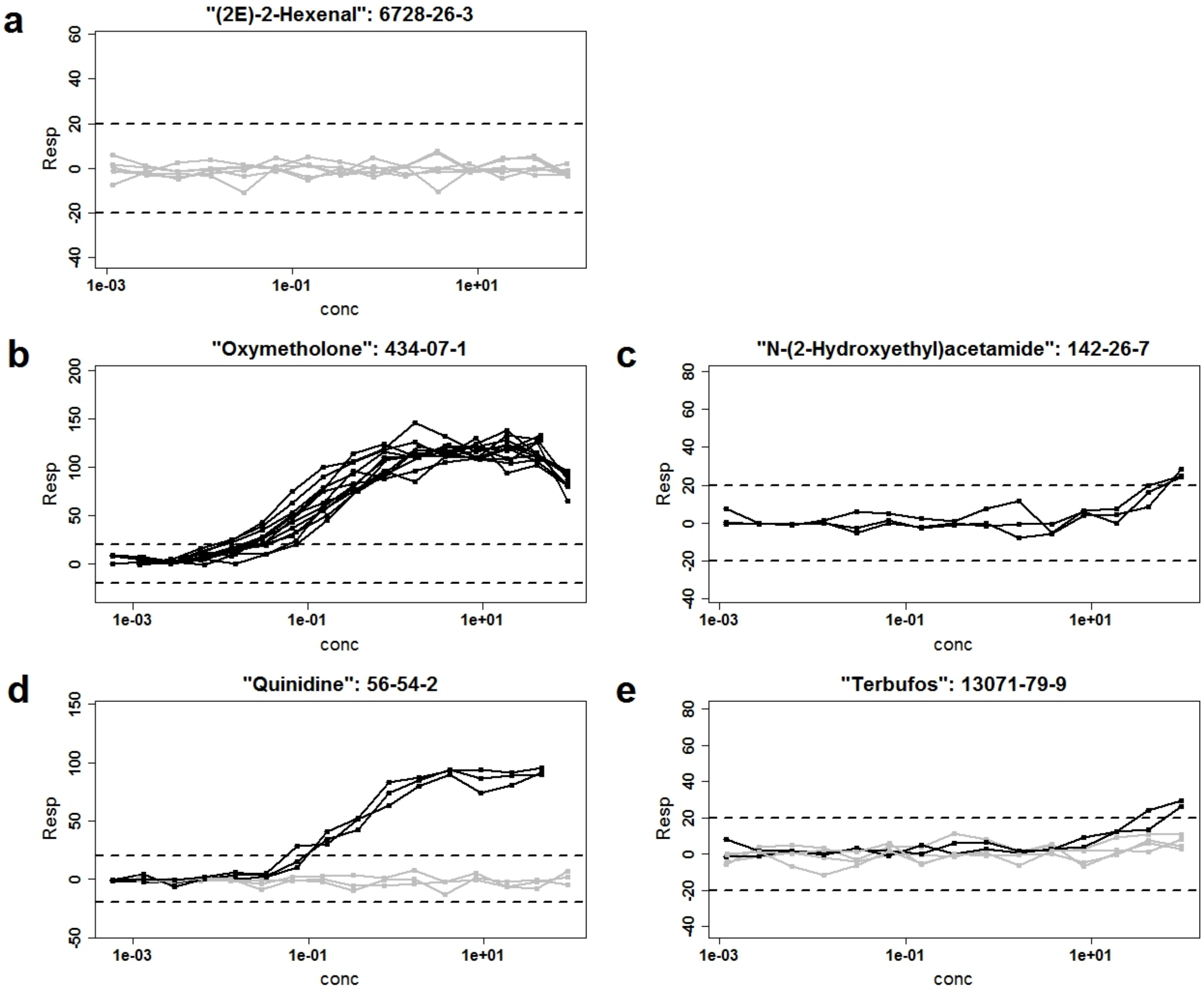

Supplementary Figure 3. Five outcome classes resulting from *CASANOVA*. Example compounds from the BG1 ER agonist data set are used to illustrate different classification scenarios. (a) *Case 2*, where all profiles within a compound are within the noise band. (b) *Conclusive Case 1* occurs when all profiles are found in a single detectable cluster. (c) *Inconclusive Case 1* refers to a single cluster for which all profiles have at least one response value lying outside of the noise band, but the response values observed outside the noise band are not significantly different from noise. (d) *Conclusive Case 3* compounds represent at least two clusters that extend outside the noise band, or one cluster inside the noise region and one cluster extending outside the noise region, when the response values outside the noise band are significantly different from the detection band. (e) *Inconclusive Case 3* compounds have at least two clusters extending outside the noise band, or one cluster inside the noise region and one cluster extending outside the noise region, but the response values observed outside the noise band are not statistically different from noise based on the one-sample t-test ($p<0.05$).

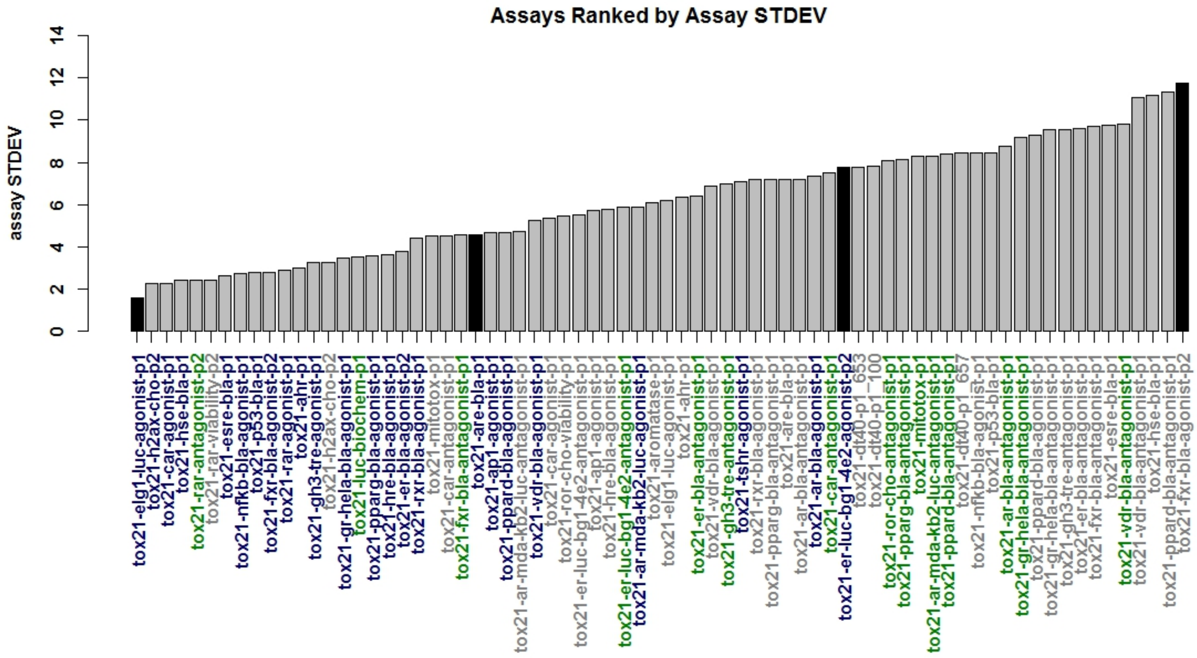

Supplementary Figure 4. Assay noise level across 72 assays based on DMSO negative control wells. The assay noise level (*STDEV*) based on the DMSO negative control wells is given on the y-axis and the assay name is on the x-axis. The assay names are dark blue (agonist), green (antagonist/inhibitor) and gray (viability). Black colored bars indicate the assays selected across the range of the assay noise levels as prototypes for simulation studies.

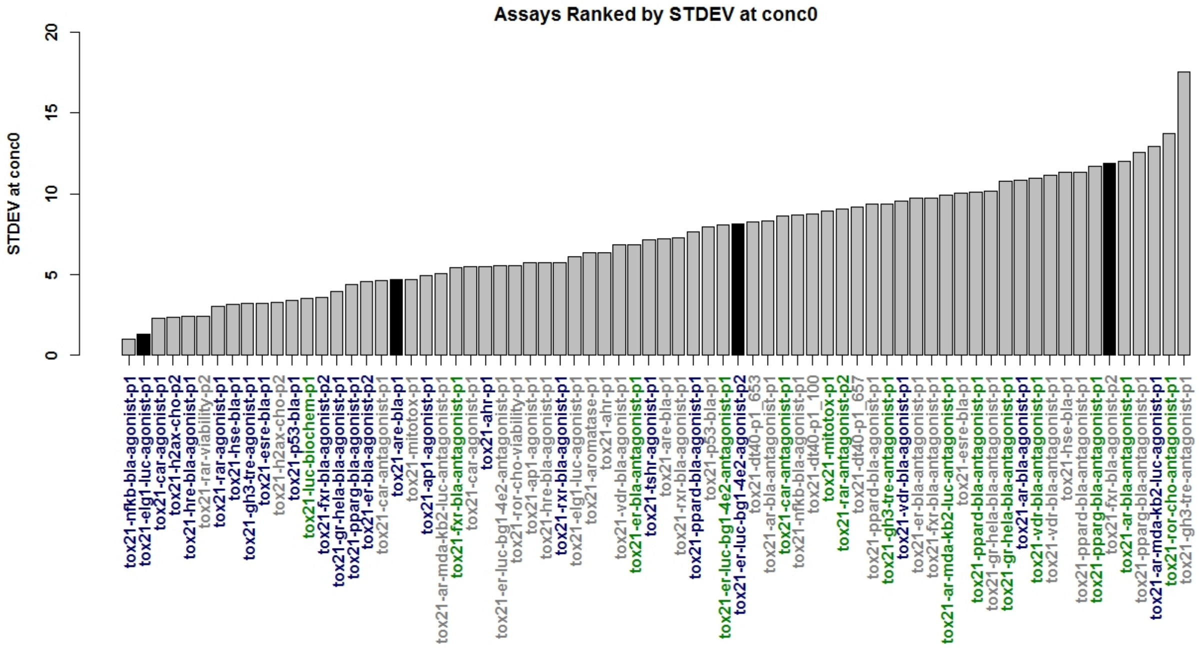

Supplementary Figure 5. Assay noise level across 72 assays based on the lowest tested concentration. The assay noise level (*STDEV*) based on the normalized responses at the lowest tested concentration (*conc0*) is given on the y-axis and the assay name is on the x-axis. The assay names are dark blue (agonist), green (antagonist/inhibitor) and gray (viability). Black colored bars indicate the assays selected across the range of the assay noise levels as prototypes for simulation studies.

Supplementary Table 1. Proportion of compounds with *N* suppliers

	Dataset 1[a]	Dataset 2[b]	Dataset 3[c]	Dataset 4[d]
N=1	0.815	0.832	0.815	0.832
N=2	0.164	0.153	0.164	0.153
N=3	0.020	0.015	0.020	0.015
N=4	0.001		0.001	

[a]*tox21-elg1-luc-agonist*, [b]*tox21-are-bla-p1*,
[c]*tox21-er-luc-bg1-4e2-agonist-p2*, [d]*tox21-fxr-bla-agonist-p2*

Supplementary Table 2. Proportion of compounds with *N* replicates per supplier

	Dataset 1[a]	Dataset 2[b]	Dataset 3[c]	Dataset 4[d]
N=3	0.937	0.936	0.937	0.936
N=6	0.052	0.051	0.052	0.051
N=9	0.003	0.003	0.003	0.003
N=12	$1.99x10^{-4}$	$2.25x10^{-4}$	$1.99x10^{-4}$	$2.25x10^{-4}$
N=42	0.006		0.006	
N=45	0.002		0.002	
N=48	$3.99x10^{-4}$	0.007	$3.99x10^{-4}$	0.007
N=51		0.002		0.002
N=54		$5.62x10^{-4}$		$5.62x10^{-4}$

[a]*tox21-elg1-luc-agonist*, [b]*tox21-are-bla-p1*,
[c]*tox21-er-luc-bg1-4e2-agonist-p2*, [d]*tox21-fxr-bla-agonist-p2*

Supplementary Table 3. Median parameters across detectable compounds

Dataset	DMSO noise σ	Lowest conc noise σ	Median Response Range	Median $\sqrt{MSE}$	Median CV
Dataset 1[a]	1.6	1.287	10.479	1.504	0.156
Dataset 2[b]	4.58	4.670	43.324	6.794	0.169
Dataset 3[c]	7.75	8.143	56.139	7.928	0.147
Dataset 4[d]	11.74	8.237	18.990	3.728	0.203

[a]*tox21-elg1-luc-agonist*, [b]*tox21-are-bla-p1*,
[c]*tox21-er-luc-bg1-4e2-agonist-p2*, [d]*tox21-fxr-bla-agonist-p2*

Supplementary Table 4. Summary of parameters in simulation study (10-fold AC_{50} differences)

Dataset Structure	Dataset 1[a]	Dataset 2[b]	Dataset 3[c]	Dataset 4[d]
Range N.clust	(1, 4)	(1, 3)	(1, 4)	(1, 3)
Mean N.clust	1.2	1.2	1.2	1.2
SD N.clust	0.5	0.4	0.5	0.4
Range N.rep/clust	(3, 48)	(3, 54)	(3, 48)	(3, 54)
Mean N.rep/clust	3.5	3.6	3.5	3.6
SD N.rep/clust	3.8	4.6	3.8	4.6
% Hill only	45.1	45.6	45.1	45.6
% Gain-Loss only	45.1	45.5	45.1	45.5
% Both models	9.8	8.9	9.8	8.9
Hill Model				
Range $log_{10}AC_{50}$	(-4.6, 2.7)	(-4.8, 2.7)	(-4.6, 2.7)	(-4.4, 2.3)
Mean $log_{10}AC_{50}$	-1.0	-1.0	-1.0	-1.0
SD $log_{10}AC_{50}$	2.0	2.0	2.0	2.0
Range *RMAX*	(-0.9, 16.2)	(-3.6, 67.7)	(-4.5, 86.2)	(-3.0, 32.9)
Mean *RMAX*	7.5	31.9	39.9	15.1
SD *RMAX*	2.3	9.5	12.3	4.4
Range *h*	(1.6E-05, 13.3)	(5.4E-06, 13.8)	(1.6E-05, 13.3)	(5.4E-06, 13.8)
Mean *h*	2.5	2.5	2.5	2.5
SD *h*	1.9	1.9	1.9	1.9
Gain-Loss Model				
Range $log_{10}AC_{50,ga}$	(-4.6, 2.7)	(-4.6, 2.6)	(-4.6, 2.7)	(-4.3, 2.3)
Mean $log_{10}AC_{50,ga}$	-1.0	-1.0	-1.0	-1.0
SD $log_{10}AC_{50,ga}$	2.0	2.0	2.0	2.0
Range $log_{10}AC_{50,la}$	(-3.6, 3.7)	(-3.5, 3.6)	(-3.6, 3.7)	(-3.3, 3.3)
Mean $log_{10}AC_{50,la}$	1.0	1.0	1.0	1.0
SD $log_{10}AC_{50,la}$	1.5	1.5	1.5	1.5
Range *RMAX*	(-0.8, 16.4)	(-3.5, 69.7)	(-4.0, 87.2)	(-2.5, 33.9)
Mean *RMAX*	7.5	31.9	40.0	15.1
SD *RMAX*	2.3	9.5	12.3	4.3
Range h_{ga}	(2.6E-05, 13.8)	(3.1E-06, 14.2)	(2.6E-05, 13.8)	(3.1E-06, 14.2)
Mean h_{ga}	2.5	2.5	2.5	2.5
SD h_{ga}	1.9	1.9	1.9	1.9
Range h_{la}	(8.6E-05, 16.8)	(1.9E-05, 16.0)	(8.6E-05, 16.8)	(1.9E-05, 16.0)
Mean h_{la}	2.5	2.5	2.5	2.5
SD h_{la}	1.9	1.9	1.9	1.9

[a]*tox21-elg1-luc-agonist*, [b]*tox21-are-bla-p1*, [c]*tox21-er-luc-bg1-4e2-agonist-p2*, [d]*tox21-fxr-bla-agonist-p2*

Supplementary Table 5. Summary of parameters in simulation study (100-fold AC_{50} differences)

Dataset Structure	Dataset 1[a]	Dataset 2[b]	Dataset 3[c]	Dataset 4[d]
Range N.clust	(1, 4)	(1, 3)	(1, 4)	(1, 3)
Mean N.clust	1.2	1.2	1.2	1.2
SD N.clust	0.5	0.4	0.5	0.4
Range N.rep/clust	(3, 48)	(3, 54)	(3, 48)	(3, 54)
Mean N.rep/clust	3.5	3.6	3.5	3.6
SD N.rep/clust	3.8	4.6	3.8	4.6
% Hill only	45.2	45.5	45.2	45.5
% Gain-Loss only	45.1	45.7	45.1	45.7
% Both models	9.8	8.8	9.8	8.8
Hill Model				
Range $log_{10}AC_{50}$	(-4.6, 2.7)	(-4.7, 2.7)	(-4.6, 2.7)	(-4.7, 2.7)
Mean $log_{10}AC_{50}$	-1.0	-1.0	-1.0	-1.0
SD $log_{10}AC_{50}$	2.2	2.2	2.2	2.2
Range *RMAX*	(-1.0, 16.8)	(-9.1, 70.2)	(-5,0, 89.2)	(-5.8, 33.9)
Mean *RMAX*	7.5	31.8	40.0	15.1
SD *RMAX*	2.3	9.4	12.2	4.3
Range *h*	(5.0E-05, 14.9)	(3.6E-05, 13.5)	(5.0E-05, 14.7)	(3.6E-05, 13.5)
Mean *h*	2.5	2.5	2.5	2.5
SD *h*	1.9	1.9	1.9	1.9
Gain-Loss Model				
Range $log_{10}AC_{50,ga}$	(-4.7, 2.7)	(-4.7, 2.6)	(-4.7, 2.7)	(-4.7, 2.6)
Mean $log_{10}AC_{50,ga}$	-1.0,	-1.0	-1.0	-1.0
SD $log_{10}AC_{50,ga}$	2.2	2.2	2.2	2.2
Range $log_{10}AC_{50,la}$	(-2.6, 3.7)	(-2.6, 3.7)	(-2.6, 3.7)	(-2.6, 3.7)
Mean $log_{10}AC_{50,la}$	1.4	1.4	1.4	1.4
SD $log_{10}AC_{50,la}$	1.5	1.5	1.5	1.5
Range *RMAX*	(-0.5, 16.4)	(-4.6, 66.7)	(-2.3, 87.3)	(-2.5, 32.1)
Mean *RMAX*	7.5	31.8	39.9	15.1
SD *RMAX*	2.3	9.5	12.2	4.4
Range h_{ga}	(2.8E-05, 13.5)	(7.9E-06, 16.8)	(2.8E-05, 13.5)	(7.9E-06, 16.8)
Mean h_{ga}	2.5	2.5	2.5	2.5
SD h_{ga}	1.9	1.9	1.9	1.9
Range h_{la}	(3.72E-05, 14.2)	(1.6E-05, 14.5)	(3.7E-05, 14.2)	(1.7E-05, 14.5)
Mean h_{la}	2.5	2.5	2.5	2.5
SD h_{la}	1.9	1.9	1.9	1.9

[a]*tox21-elg1-luc-agonist*, [b]*tox21-are-bla-p1*, [c]*tox21-er-luc-bg1-4e2-agonist-p2*, [d]*tox21-fxr-bla-agonist-p2*

Supplementary Table 6. Bias and variance of $log_{10}AC_{50}$ parameter for Hill model curves (10% error)

True AC_{50}	*True RMAX*	*n*	*AVG*	*MEDIAN*	*WT AVG*	*ONE MODEL*
			Bias (and variance) of $log_{10}AC_{50}$			
**1.00e-03 *UPPER PLATEAU ONLY***	25	3	0.45 (15.58)	0.16 (7.14)	0.01 (6.15)	1.39 (21.29)
	25	6	0.53 (7.96)	0.13 (1.47)	0.06 (1.15)	1.45 (20.16)
	25	9	0.41 (5.84)	0.03 (0.45)	0.17 (0.78)	1.61 (21.33)
	25	12	0.54 (3.91)	0.04 (0.19)	0.13 (0.12)	1.37 (16.41)
	50	3	1.20 (4.36)	0.41 (2.54)	0.05 (2.65)	0.46 (3.17)
	50	6	1.22 (2.16)	0.23 (0.73)	0.05 (0.58)	0.42 (3.11)
	50	9	1.21 (1.42)	0.08 (0.17)	0.05 (0.41)	0.43 (3.15)
	50	12	1.21 (1.09)	0.06 (0.08)	0.02 (0.17)	0.42 (2.84)
	100	3	0.29 (0.50)	0.08 (0.25)	0.03 (0.21)	0.06 (0.12)
	100	6	0.25 (0.21)	0.03 (0.02)	0.05 (0.16)	0.04 (0.04)
	100	9	0.28 (0.16)	0.02 (0.01)	0.07 (0.08)	0.05 (0.07)
	100	12	0.26 (0.11)	0.02 (0.01)	0.05 (0.09)	0.04 (0.05)
**0.1 *UPPER AND LOWER PLATEAUS***	25	3	0.42 (12.13)	0.24 (5.34)	0.02 (2.18)	0.06 (9.40)
	25	6	0.34 (5.48)	0.06 (0.55)	0.01 (0.60)	0.20 (8.05)
	25	9	0.40 (3.92)	0.03 (0.11)	0.01 (0.47)	0.08 (8.44)
	25	12	0.41 (2.88)	0.02 (0.07)	0.02 (0.17)	0.14 (7.62)
	50	3	0.04 (1.37)	* (0.05)	0.01 (0.04)	* (0.03)
	50	6	0.08 (0.75)	0.01 (0.02)	* (0.02)	* (0.03)
	50	9	0.08 (0.44)	* (0.02)	0.01 (0.02)	0.02 (0.27)
	50	12	0.15 (0.30)	* (0.01)	* (0.01)	* (0.03)
	100	3	* (0.01)	* (0.01)	* (0.01)	* (0.01)
	100	6	* (*)	* (*)	* (*)	* (0.01)
	100	9	* (*)	* (*)	* (*)	* (0.01)
	100	12	* (*)	* (*)	* (*)	* (0.01)
**10 *LOWER PLATEAU ONLY***	25	3	1.36 (11.05)	0.79 (7.32)	0.08 (5.96)	2.10 (19.64)
	25	6	1.20 (6.27)	0.50 (2.34)	0.05 (1.33)	2.34 (22.69)
	25	9	1.17 (4.31)	0.26 (1.01)	0.14 (1.89)	2.13 (35.03)
	25	12	1.27 (3.31)	0.28 (0.69)	0.05 (0.32)	2.02 (19.22)
	50	3	1.90 (4.65)	1.05 (5.22)	0.37 (3.51)	0.74 (5.32)
	50	6	1.94 (2.45)	0.75 (1.96)	0.29 (1.83)	0.76 (5.27)
	50	9	1.91 (1.62)	0.41 (0.96)	0.22 (1.25)	0.75 (4.90)
	50	12	1.87 (1.09)	0.35 (0.48)	0.27 (0.76)	0.79 (5.47)
	100	3	0.75 (1.10)	0.30 (0.76)	0.15 (0.98)	0.11 (0.25)
	100	6	0.77 (0.58)	0.21 (0.21)	0.16 (0.84)	0.10 (0.20)
	100	9	0.73 (0.36)	0.13 (0.09)	0.22 (0.84)	0.10 (0.17)
	100	12	0.76 (0.28)	0.13 (0.05)	0.23 (0.59)	0.09 (0.12)

Values of bias or variance less than 0.01 are indicated by "*".
